# Origin of land plants: Do conjugating green algae hold the key?

**DOI:** 10.1186/1471-2148-11-104

**Published:** 2011-04-18

**Authors:** Sabina Wodniok, Henner Brinkmann, Gernot Glöckner, Andrew J Heidel, Hervé Philippe, Michael Melkonian, Burkhard Becker

**Affiliations:** 1 Biozentrum Köln, Botanik, Universität zu Köln, Zülpicher Straße 47b, 50674 Köln, Germany; 2 Centre Robert-Cedergren, Département de Biochimie, Université de Montréal, Succursale Centre-Ville, Montréal, Qc H3C3J7, Canada; 3 Leibniz-Institute of Freshwater Ecology and Inland Fisheries, IGB Müggelseedamm 310, D-12587 Berlin, Germany; 4 Fritz-Lipmann-Institut, Beutenbergstraße 11, 07745 Jena, Germany

## Abstract

**Background:**

The terrestrial habitat was colonized by the ancestors of modern land plants about 500 to 470 million years ago. Today it is widely accepted that land plants (embryophytes) evolved from streptophyte algae, also referred to as charophycean algae. The streptophyte algae are a paraphyletic group of green algae, ranging from unicellular flagellates to morphologically complex forms such as the stoneworts (Charales). For a better understanding of the evolution of land plants, it is of prime importance to identify the streptophyte algae that are the sister-group to the embryophytes. The Charales, the Coleochaetales or more recently the Zygnematales have been considered to be the sister group of the embryophytes However, despite many years of phylogenetic studies, this question has not been resolved and remains controversial.

**Results:**

Here, we use a large data set of nuclear-encoded genes (129 proteins) from 40 green plant taxa (Viridiplantae) including 21 embryophytes and six streptophyte algae, representing all major streptophyte algal lineages, to investigate the phylogenetic relationships of streptophyte algae and embryophytes. Our phylogenetic analyses indicate that either the Zygnematales or a clade consisting of the Zygnematales and the Coleochaetales are the sister group to embryophytes.

**Conclusions:**

Our analyses support the notion that the Charales are not the closest living relatives of embryophytes. Instead, the Zygnematales or a clade consisting of Zygnematales and Coleochaetales are most likely the sister group of embryophytes. Although this result is in agreement with a previously published phylogenetic study of chloroplast genomes, additional data are needed to confirm this conclusion. A Zygnematales/embryophyte sister group relationship has important implications for early land plant evolution. If substantiated, it should allow us to address important questions regarding the primary adaptations of viridiplants during the conquest of land. Clearly, the biology of the Zygnematales will receive renewed interest in the future.

## Background

The ancestors of modern land plants (embryophytes) colonized the terrestrial habitat about 500 to 470 million years ago (Ordovician period [1-3]). This event was undoubtedly one of the most important steps in the evolution of life on earth [4-6], thereby establishing the path to our current terrestrial ecosystems [7] and significantly changing the atmospheric oxygen concentration [8,9]. Since this time three major groups of land plants evolved: bryophytes (liverworts, hornworts and mosses), pteridophytes (lycophytes and monilophytes) and spermatophytes with the latter dominating most habitats today.

It is widely accepted that embryophytes evolved from green algae, or more specifically, from a small but diverse group of green algae known as the streptophyte algae (charophycean algae). Streptophyte algae and embryophytes together constitute the division Streptophyta, which likely split from the Chlorophyta (all other green algae) about 725-1200 MY ago [10-12]. Streptophyta and Chlorophyta comprise the Viridiplantae, one of the three evolutionary lineages derived from the single primary endosymbiosis of a cyanobacterium and a eukaryotic host cell [13].

The Streptophyta are characterized by several morphological (e.g., structure of flagellate reproductive cells, if present [14]), and physiological characters (e.g., occurrence of glyceraldehyde-3-phosphate dehydrogenase isoform B, GAPDH B [15], leaf peroxisome type of photorespiration [16,17]). Furthermore, several typical embryophyte traits have evolved within the streptophyte algae (e.g., cell division using a phragmoplast, structure of the cellulose synthase complex [4]). However, the streptophyte algae differ greatly in cellular organization and reproduction. Molecular phylogenies indicate that the Mesostigmatales and Chlorokybales form a clade that is a sister-group to all other streptophytes, currently containing only two genera: the biflagellate *Mesostigma *and the sarcinoid (non-motile cells occurring in packages of four) *Chlorokybus *[18,19]. The Klebsormidiales, which is comprised of filamentous algae [14], is the sister group to the remaining streptophyte algae and the embryophytes. The phylogenetic position of the other three groups of streptophyte algae is currently controversial. The conjugating green algae (Zygnematales) today represent the most species-rich group of streptophyte algae and are characterized by their unique mode of sexual reproduction. They have completely lost flagellate cells, using instead conjugation for sexual reproduction [20]. The conjugating green algae include both filamentous and unicellular forms. The last two groups of streptophyte algae, the Coleochaetales and Charales, are filamentous with apical growth and an oogamous mode of sexual reproduction. Based on morphological complexity, either of the latter two groups have been suggested to be the sister group of the embryophytes [4]. In many illustrations referring to the evolution of streptophyte algae and embryophytes in textbooks [e.g. [21]] or review articles [14,22,23], the Charales (stoneworts) are depicted as the sister group of the embryophytes. The strongest support for a sister group relationship between Charales and embryophytes was obtained in a phylogenetic analysis using four genes (*atpB *and *rbcL *[plastid], *nad5 *[mitochondria], and SSU RNA [nuclear] using 26 streptophyte algae, eight embryophytes, and five chlorophytes and one glaucophyte as outgroup [24]). In contrast, analyses using plastid LSU and SSU ribosomal RNAs or whole chloroplast genomes support the Zygnematales or a clade consisting of Zygnematales and Coleochaetales as sister group of the embryophytes [25-27].

Here we use ESTs from six different streptophyte algae for a phylogenomic analysis including 21 embryophytes. We show that the Charales are most likely not the sister group of the embryophytes, instead our analyses indicate that either the conjugating green algae or less likely a sister group formed by *Coleochaete *and the Zygnematales might be the closest living relatives of embryophytes, in agreement with previous phylogenetic analyses based on chloroplast genomes.

## Results

### Zygnematales alone or together with Coleochaetales as the sister group of embryophytes

New ESTs were sequenced from the streptophyte algae *Klebsormidium subtile*, *Coleochaete scutata*, and *Chara vulgaris *and the chlorophyte alga *Pyramimonas parkeae *(see Material and Methods for details). We assembled a data set of 129 expressed genes (30,270 unambiguously aligned amino acid positions) for 40 viridiplant taxa including six streptophyte algae (*Mesostigma*, *Klebsormidium*, *Chara*, *Coleochaete*, *Closterium *and *Spirogyra*) using the chlorophytes as outgroup to root the trees.

The data set was analyzed by maximum likelihood (ML) and Bayesian inference (BI) methods using several evolutionary models. We first evaluated the fits of the models to our data set using cross validation (Table 1). The site-heterogeneous CATGTR model is the best of the four models under study. The site-heterogeneous CAT model, which assumes uniform exchangeability rates among amino acids, has a much better fit than the site-homogeneous LG+F and GTR models, and is just slightly worse than the CATGTR model. Interestingly, the data set is sufficiently large to accurately estimate the amino acid exchangeability rates, since the GTR model has a better fit to the data than the LG+F model, where these parameters were learned from numerous alignments [28]. The simplifying assumption of equal rates of the CAT model, albeit biologically unsound and rejected by cross validation (in favor of the CATGTR model), has the advantage of allowing a significant increase in computational speed [29], and was therefore used for bootstrap analysis.

**Table 1:** Cross-validation results for the data set of 40 viridiplant species and 30,270 positio ns (a positive score indicates a better fit)

Model compared	Likelihood difference (±SD)
GTR+Γ4 vs LG+Γ4	377.99 ± 28.51
CAT+Γ4 vs LG+Γ4	1187.27 ± 78.75
CATGTR+Γ4 vs LG+Γ4	1547.08 ± 63.35

**Figure 1:**
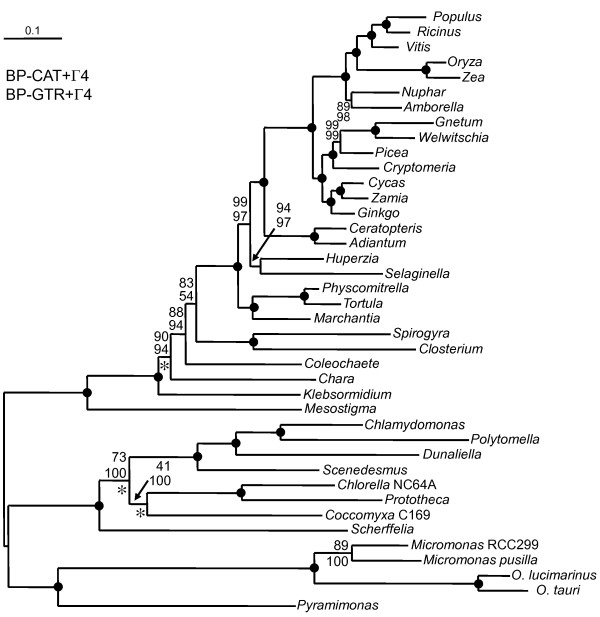
**Consensus Tree inferred by PhyloBayes under the CAT+ Γ4 using the viridiplant data set of 40 taxa and 30,270 amino acid positions (129 concatenated nuclear encoded proteins)**. An identical topology was obtained with two different methods (ML, BI) and four different models applied (site homogeneous ML, LG+F+Γ4 and GTR+Γ4; site heterogeneous BI, CAT+Γ4 and CATGTR+Γ4). Numbers represent (in order from top to bottom) the bootstrap support values for the PhyloBayes CAT+Γ4 and the RAxML GTR+Γ4 analyses. Black dots indicate that the branch was supported by a BP of 100% using both models. All except three nodes, which are indicated by a star, were supported by posterior probabilities (PP) of 1. The scale bar denotes the estimated number of amino acid substitutions per site.

Despite very different model fits, the same tree topology (Figure 1) was obtained in all analyses. Bootstrap support values were computed for both methods, using the site homogeneous GTR+Γ4 model (ML) and the site heterogeneous CAT+Γ4 model (BI). The posterior probabilities of all nodes for both the CAT+Γ4 and CATGTR+Γ4 models were 1 except for three nodes (0.99 each, indicated with an asterisk in Figure 1). The molecular phylogeny of embryophytes and chlorophyte algae (outgroup) is in agreement with other recently published phylogenies [14,23,30-32] and supports the monophyly of liverworts and mosses which is however still a matter of debate [33]. The phylogeny of the streptophyte algae is asymmetrical. *Mesostigma *is sister to all the remaining streptophytes, as in other studies without *Chlorokybus *[18,19,24]. There is a long, highly supported, branch at the base of the clade uniting the other streptophyte algae and embryophytes, likely indicating the elapse of a substantial amount of time. In contrast, the phylogeny of the remaining streptophyte algae resembles an adaptive radiation, with the five major lineages, including the embryophytes, appearing serial, but with relatively short internal branches. Within this clade, *Klebsormidium *is sister to all the other species. The Charales are sister to a clade comprising Coleochaetales, Zygnematales and embryophytes and are therefore an unlikely candidate as the sister-group of the embryophytes (only BP of 5% and 2% with CAT+Γ4 and GTR+Γ4 models, respectively). The latter clade is moderately well supported (BP of 88% and 94%) and its support is lower than for the clade *Klebsormidium*+Chara+Coleochaetales+Zygnematales+embryophytes (100%). However, the sister-group relationship of embryophytes and Zygnematales (BP 83% and 54%) is less well supported, especially in the analysis under the site-homogeneous GTR+Γ4 model. A fraction of the bootstrap replicates supports the alternative topology that unites *Coleochaete *with the Zygnematales (BP 12% and 35%, respectively). The data indicate that *Mesostigma*, *Chara*, *Klebsormidium *and *Coleochaete *are evolving at a comparable and moderate rate, with embryophytes and Zygnematales evolving faster. For instance, the Zygnematales appear to have evolved twice as fast as *Coleochaete*.

**Figure 2:**
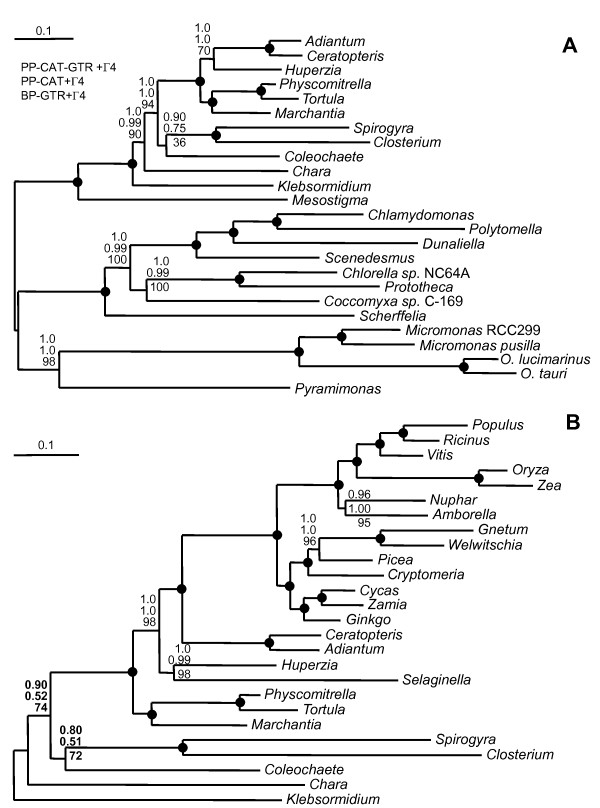
**Consensus Tree inferred by PhyloBayes using reduced data sets of 25 taxa (A. Spermatophytes and *Selaginella *eliminated) or 26 taxa (B. Chlorophytes and *Mesostigma *eliminated)**. The same methods and models were used as in Figure 1, with the only exception that no bootstrap analyses were performed for the PhyloBayes analyses, for which only the posterior probabilities are given. The alternative taxon samplings were aimed at either eliminating the fast evolving embryophytes (all spermatophytes and *Selaginella*) (**A**) or the distantly-related outgroup sequences eliminating chlorophytes and *Mesostigma *(**B**).

This difference in evolutionary rate suggests that the grouping of embryophytes and Zygnematales could be due to a long-branch attraction (LBA) artifact [34]. To explore this possibility, we analyzed two reduced taxon samples, where the 15 fastest-evolving land plants have been discarded (Figure 2A) and the 13 long-branched chlorophytes and *Mesostigma *were not used as an outgroup (Figure 2B). The impact of LBA should be reduced in both cases. Again, the four models and the two methods (ML and BI) lead to identical topologies for both data sets. Interestingly, in both cases, the topology differs from the one of the complete data set (40 species, Figure 1) by the appearance of a sister-group relationship between *Coleochaete *and Zygnematales. However, this grouping receives non-significant support (PP between 0.51 and 0.90 and BP of 36 and 72%). In both cases, the Zygnematales plus *Coleochaete *clade is the closest relative to embryophytes (however, with limited support when the outgroups were removed [BP of 74%, Figure 2B]). It is noteworthy, that in none of the analyses was *Chara *recovered as a sister clade to the embryophytes (low BP: 2% in Figure 2A and 19% in Figure 2B).

**Figure 3:**
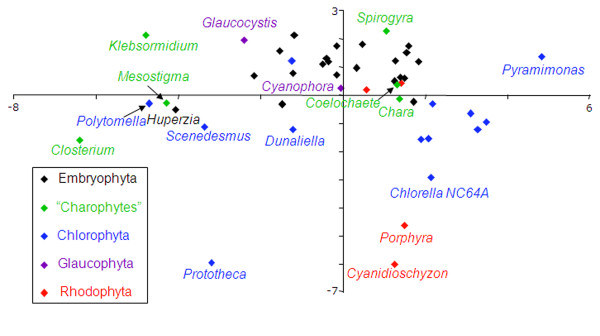
**Principal component analysis of the complete 46 taxa data set**. The two first axes of the multidimensional space are shown, they account together for 48% of the data. The principal component analysis demonstrates that the majority of the sequences have a homogeneous amino acid composition. Nevertheless, there are also several outliers most of them expectedly associated with distant outgroup species; more precisely there are two red algae, several chlorophytes, but also three streptophyte algae (*Mesostigma*, *Klebsormidium *and *Closterium*) and the embryophyte *Huperzia*.

Another cause of systematic errors in phylogenetic inference is the compositional heterogeneity across taxa [35,36]. A principal component analysis of the amino acid composition (Figure 3) demonstrates that *Coleochaete*, *Chara *and *Spirogyra *and most embryophytes have a similar composition. Other organisms (e.g. *Prototheca*, *Chlorella, Closterium *and *Klebsormidium*) show much larger compositional differences, but are correctly placed in a phylogenetic tree due to the presence of a strong phylogenetic signal, as the strongly supported monophyly of *Closterium*+*Spirogyra *and *Selaginella+Huperzia *illustrate. We explored the potential impact of compositional heterogeneity on our inference by using the Dayhoff recoding, an approach known to be efficient [37-39]. Interestingly, the three models (GTR, CAT and CATGTR) and the three taxon samples in Figures 1 and 2 A, B all lead to the same topology as in Figure 1. Since the Dayhoff recoding reduces not only compositional heterogeneity but also saturation, the sister-group relationship between the Zygnematales and embryophytes as observed in Figure 1 is less likely to result from systematic error.

## Discussion

Previous studies of the phylogeny of Streptophyta were restricted mainly to ribosomal RNA or sequences of organellar origin [24-27]. We now, used for the first time, large data sets of nuclear-encoded proteins for phylogenetic studies in this important evolutionary lineage. Our phylogenetic analyses are in agreement with both phylogenies obtained using a data set of concatenated plastid proteins or ribosomal RNAs [26,27], but are in conflict with the 4 gene tree mentioned above [24]. In contrast, *Coleochaete *was found to be sister to embryophytes [40] in a recent analysis based on 77 nuclear encoded ribosomal proteins (12,459 amino acid positions). However, as this study failed to recover the monophyly of the Coleochaetales (placing *Chaetosphaeridium *within the Zygnematales) the conclusions from this study should be treated with caution. In the 4 gene analysis the topology (Zygnematales, (Coleochaetales, (Charales, embryophytes))) was observed. This analysis suggested that the streptophyte algae regularly (without reversions) evolved towards increasing morphological complexity (resulting in a larger number of shared morphological characters with embryophytes). In contrast, our results and the results obtained using chloroplast data [25,26] suggest that most likely the morphologically simpler Zygnematales (or a clade consisting of Zygnematales and Coleochaetales) is the sister group of embryophytes, rather than the Charales. It seems plausible that the simpler morphology of extant Zygnematales represents a secondary simplification, similar to the loss of flagellate cells in this group, which may actually represent an adaptation to ensure sexual reproduction in the absence of free water [41]. Alternatively, the morphological complexity of the Charales and Coleochaetales might have evolved independently after the three evolutionary lines (Coleochaetales, Charales, and Zygnematales) diverged. This kind of scenario was already proposed by Stebbins and Hill [41]: They suggested that the early evolution of streptophyte algae took place in a moist terrestrial habitat and involved rather simple unicellular types. They considered the extant Coleochaetales, Charales, Klebsormidiales and Zygnematales to be derived forms with a secondary aquatic life style. A fast initial radiation at the time of colonization of the terrestrial habitat by the ancestors of modern streptophyte algae as proposed by Stebbins and Hill [41] may also explain the relative difficulty to infer the phylogenetic relationships among the four groups of streptophyte algae, enhanced by the accelerated evolutionary rate of the Zygnematales. However, in contrast to Stebbins and Hill [41], we argue that at least some of the morphological complexity had evolved prior to the early radiation of the streptophyte algae for the following reasons: (1) Based on the available fossil record, the Charales already had a morphology similar to that of extant forms in the Silurian period [42], (2) the available EST data indicate that Zygnematales, Coleochaetales and Charales possess homologues of a number of proteins that are involved in the development of morphologically complex structures in embryophytes, such as GNOM, Wuschel, Meristemlayer 1, MIKc-type MADS-box protein ([43,44] and unpublished results). Taken together, these data lend support to the idea that the extant morphological complexity of the Charales and Coleochaetales is an ancient trait that may have been secondarily lost in the Zygnematales.

Alternatively, as was found by the comparison of *Volvox*, a multicellular system, with its close relative *Chlamydomonas reinhardtii*, the evolution of multi-cellular structures seems to rely mainly on the reorganization and differential regulation of already existing genes [45]. From this point of view, the complex morphologies of the Charales and Coleochaetales could have evolved completely independently by using the toolbox already present in the common ancestor of these streptophyte algae. Although our results, based on the complete data set, favor the Zygnematales as the sister group of the embryophytes, the results from the two alternative taxon sampling tests, in which we tried to reduce as much as possible potential disturbing influences of the LBA artifacts, seem to point rather to a sister group relationship between *Coleochaete *and the Zygnematales. In contrast, Dayhoff coding favors the grouping of the Zygnematales with the embryophytes, whatever the taxon sampling. Since this recoding is expected to reduce several sources of systematic errors, this topology is more likely. However, the support in this part of the tree remains limited, and large-scale genomic data from more streptophyte algae (especially Coleochaetales, Charales and Klebsormidiales) are needed to resolve this question. Whatever the relative position of *Coleochaete *and the Zygnematales, our analysis supports the scenario of a secondary loss of morphological complexity in the Zygnematales.

The first land plants encountered a more extreme environment compared to a freshwater habitat, with large fluctuations in water content (wetting and desiccation), radiation intensity (visible light and UV) and nutrient supply. Potentially, the last common ancestor of the Zygnematales and embryophytes was better adapted to these types of environmental stressors than other streptophyte algae. The more variable environmental conditions might have also favored the evolution of more complex signaling pathways [46]. Rensing et al. [46] discussed several proteins likely to be important for the adaptation of embryophytes to their terrestrial habitat. Preliminary analysis of the available ESTs from streptophyte algae indicate that expressed genes similar to most of the proteins listed by Rensing et al. [46] can be found in various streptophyte algae (Table 2). For example, proteins similar to major light harvesting complex II proteins (lhcb1-3), which were considered to be missing from green algae [46-48], are clearly found in ESTs from streptophyte algae except *Mesostigma *(Table 2). Expressed genes similar to late embryo abundant (LEA) proteins known to protect spermatophyte seeds from desiccation [49] are found in several streptophyte algae (Table 2).

**Table 2:** Proteins proposed to be important in the adaptation to the terrestrial habitat [46] are present in streptophyte algae.

	Gene name	TAIR number	*Klebsormidum subtile*	*Chara vulgaris*	*Coleochaete scutata*	*Coleochaete* *orbicularis*	***Closterium spec***.	*Spirogyra pratense*
Photosynthesis	Lhcb3	At5g54270	8 (e-119)	9 (e-38)	3 (e-117)	78 (e-102	(e-126)	37 (e-126)

Dessication tolerance	Lea1^1)^	At3G51810	1 (e-20)	1 (e-33)	1(e-19)	1 (e-189	n.d.	?

Ethylen signaling	EIN	At5g03280	n.d.	n.d.	n.d.	1 (e-22)	n.d.	n.d.
	ETR	At1g66340	n.d.	n.d.	n.d.	n.d.	n.d.	1 (e-105)
	ACS	At5g65800	n.d.	n.d.	n.d.	1 (e-53)	n.d.	1 (e-49)

Auxin signaling	ARF	At5g62010	n.d.	n.d.	n.d.	n.d.	n.d.	1 (e-44)
	PIN	At1g73590	n.d.	n.d.	n.d.	n.d.	n.d.	1 (e-33)
	PINOID	At2g34650	n.d.	n.d.	n.d.	n.d.	n.d.	1 (e-72)

BLAST analyses were performed to identify putative homologues in the indicated streptophyte algae. The number of contigs and the best e-value obtained are given.

n.d. not detected, ? no clear result. ^1)^for most species ESTs showing similarity to several different Lea proteins were found (e.g., in *Klebsormidium *ESTs we detected additional sequences showing similarity to Lea14, Lea76 and Lea D29)

The probable fast radiation of the derived lineages of streptophyte algae (see above) in conjunction with the secondary morphological simplification of the Zygnematales makes it difficult to find any synapomorphies for the possible sister group relationship of the Zygnematales and embryophytes. We note two complex traits that might potentially support this relationship. Firstly, components of the "auxin signaling machinery" are highly conserved in embryophytes [46,50], but appear to be absent in streptophyte algae, except for the auxin binding protein (abp1), which can be found in various green algae including chlorophytes [50]. However, as also noted by De Smet et al. [51] the recently published ESTs from *Spirogyra *[52] include expressed genes similar to components (ARF, PIN, PINOID, Table 2) of the embryophyte-specific "auxin signaling machinery". Secondly, embryophytes generally show chloroplast movements in response to high (avoidance response) or low light (accumulation response), which has been shown to be of ecological importance [53]. Chloroplast movements in response to low or high light conditions have also been reported for several Zygnematales [53] as well as for some chlorophytes, diatoms and *Vaucheria *[54]. While the photoreceptor is not known for most algae, recent work has shown that, in *Mougoetia scalaris *(Zygnematales), phototropin and neochrome are used as photoreceptor similar to the situation in *Physcomitrella *and *Adiantum *[54,55], suggestive of a common origin of this response.

## Conclusions

Knowledge of the phylogenetic relationships within streptophyte algae is of crucial importance for developing a realistic scenario for the colonization of the terrestrial habitat and the origin and early evolution of embryophytes. Phylogenomic analyses of nuclear and chloroplast data now indicate that the Charales are most likely not the closest living extant relatives of the embryophytes despite their morphological complexity. Instead, the analyses favor either the Zygnematales or, less likely, a clade consisting of the Zygnematales and Coleochaetales as the sister group of embryophytes. An extended taxon sampling and/or analyses of larger data sets such as complete genomes/transcriptomes will likely be necessary to shed further light on the elusive sister group of the embryophyte plants

## Methods

### Preparation of cDNA libraries and EST sequencing

The preparation of cDNA libraries for *Pyramimonas parkeae*, *Klebsormidium subtile *and *Coleochaete scutata*, EST sequencing and processing of the primary reads have all been described by Wodniok et al. [56]. *Chara vulgaris *zygotes were collected from the botanical garden of the Universität zu Köln. Zygotes were surface-sterilized using the following protocol (modified after [57,58]): after washing with distilled water, zygotes were rinsed with EtOH (70%, 1-2 min), followed by sodium hypochlorite (7-12%, 20-25 min). In some experiments the EtOH step was omitted. Surface-sterilized zygotes were rinsed repeatedly with sterile distilled water to remove hypochlorite and ethanol. For germination, single zygotes were each placed in a well of a microtiter plate and incubated in *Chara*-medium ([57,58]) at 24°C and a 14/10 h light dark cycle at 20 - 40 μEm^-2^s^-1^. Zygotes germinated after 4-6 weeks. After germination, young plants were transferred into 100 ml Erlenmeyer flasks containing 10 ml agar overlaid with 40 ml *Chara*-medium. Cultures often underwent sexual reproduction within one year. Preparation of cDNA libraries and Sanger sequencing was done as described earlier [56,59].

For 454 sequencing *Chara *total RNA was isolated using Trizol (Invitrogen) following the manufacturer's instructions. The cDNA library was made from the RNA with the Mint cDNA synthesis kit (Evrogen) using 21 cycles in the PCR amplification step. The cDNA subsequently was converted to a Roche/454 sequencing library (rapid) according to the manufacturer's protocols. Sequencing of this library yielded 740,341 raw reads with 245 Mb raw sequence data. Assembly resulted in 13,615 contigs spanning 6.5 Mb.

### Phylogenetic analyses

The data set assembly and the detection of possible non-orthologous sequences were performed as described elsewhere in detail [60,61]. Briefly, the latter approach is based on the assumption that the tree obtained in the phylogenetic analysis of the concatenated data set (super-matrix) is a good proxy of the "true" tree. All single gene data sets were analyzed separately with RAxML using the LG+Γ4 model including 100 bootstrap pseudo-replicates [62]. Nodes of the single gene trees that are in conflict to the reference tree (super-matrix) and that are supported by a bootstrap value ≥ 70% are considered to be incongruent. Most of these conflicts are usually due to problems of the phylogenetic reconstruction (stochastic, but also systematic errors), most often the conflict can be resolved by a nearest-neighbor-interchange (NNI). However, occasionally there are also true conflicts related to the presence of paralogous or xenologous sequences and the genes were therefore discarded from the super-matrix.

The final data set was assembled using Scafos [63] and consisted of 46 taxa, including six distant outgroups (two glaucophytes and four red algae), with a total of 30,270 amino acid positions coming from 129 genes. To reduce the amount of missing data three chimerical sequences were made within the streptophytes, two at the genus and one at the family level, as well as a higher order (class) chimera invoking the Florideophyceae. By allowing no more than 16 missing taxa for any given gene, the final amount of missing data in the supermatrix was 30%. The concatenated data set and three sub-samples with a different species sampling were analyzed with two different probabilistic methods, i.e. Maximum Likelihood (ML) as implemented in the program RAxML [62] and Bayesian Inference with PhyloBayes [64]. The RAxML analyses were done under the site-homogeneous LG+F+Γ4 and GTR+ Γ4 models, and included a fast-bootstrap analysis with 100 pseudo-replicates with the same models. The Bayesian analyses were performed under the site-heterogeneous CAT+Γ4 and CATGTR+Γ4 models [29]. Two independent chains were run per analysis for 10,000 cycles (with each 10^th ^cycle sampled) and their bipartitions were compared after the elimination of the "burn-in" in order to test the quality of the convergence. The maximal difference observed between bipartition frequencies of two independent runs was always lower than 0.1. Furthermore, a bootstrap analysis with 100 pseudo-replicates was performed under the CAT+Γ4 model, a chain per dataset was run for the same length and sampled as above, the "burn-in" was fixed to 1,000 cycles. Each of the 100 resulting consensus trees was then used as an input for the program Consense of the Phylip package to generate the bootstrap consensus tree [65]. We performed cross validation tests to evaluate the fit of the four models used (LG, GTR, CAT and CATGTR). The analysis was performed in PhyloBayes, using ten randomly generated replicates, in which the original data set was divided into training data sets (9/10 of the positions) to estimate the parameters of the given model and into the test data sets (1/10 of the positions) to calculate with these parameters the likelihood scores.

The amino-acid composition of the 46 species data set was visualized by assembling a 20 × 46 matrix containing the frequency of each amino acid per species using the program NET from the MUST package [66]. This matrix was then displayed as a two-dimensional plot in a principal component analysis, as implemented in the R package. To counteract sequence bias, we recoded the 20 amino acids into six groups as previously proposed [39]. Phylogenetic analysis of the three Dayhoff-recoded data sets was performed using PhyloBayes with the GTR+ Γ4, CAT+Γ4 and CATGTR+Γ4 models.

### Data access

EST reads (Sanger) were deposited in Genbank under the following accession numbers: *Klebsormidium subtile *(LIBEST_027068), *Coleochaete scutata *(LIBEST_027067). The 454 sequence of *Chara vulgaris *can be found in the Sequence Read Archive (SRP005673). The alignment has been deposited to Treebase (S11199).

## Authors' contributions

SW prepared the cDNA libraries from *Pyramimonas*, *Klebsormidium*, *Coleochaete *and *Chara*. HP and SW carried out the sequence alignment. GG isolated the RNA from *Chara *for 454 sequencing, sequenced the cDNA libraries and analyzed the data. AJH prepared the *Chara *cDNA for 454 sequencing. HB, HP and SW performed phylogenetic analyses. MM participated in the design of the study and analyzed the data. BB conceived the study, participated in its design, analyzed the data and wrote the draft of the manuscript. All authors read and approved the final manuscript.
